# Results of a New Treatment Protocol for Sudden Sensorineural Hearing Loss Using Betamethasone for Intratympanic Therapy

**DOI:** 10.1055/s-0044-1788779

**Published:** 2024-10-25

**Authors:** Paula Tardim Lopes, Jessica Almeida, Ricardo Ferreira Bento

**Affiliations:** 1 Department of Otorhinolaryngology, School of Medicine, Hospital das Clínicas, Universidade de São Paulo, São Paulo, SP, Brazil; 2 Department of Otology, Fundação Otorrinolaringologia, São Paulo, SP, Brazil

**Keywords:** hearing loss, sensorineural hearing loss, sudden, salvage therapy steroids, betamethasone

## Abstract

**Introduction** Sudden sensorineural hearing loss (SSNHL) is defined as a rapid sensation of hearing impairment in one or both ears. Based on its personal impact on patients, the present study advances the analysis of new treatments for the prompt recognition and management of SSNHL, with higher chances of improvements in terms of hearing recovery and quality of life of the patients.

**Objective** To describe the intratympanic (IT) corticoid infiltration technique, to demonstrate the efficacy of betamethasone injection as a sequential treatment in patients whose initial systemic steroid treatment failed, as well as to compare its use in different treatment periods.

**Methods** The present clinical trial was conducted with 37 patients referred to our office with the diagnosis of SSNHL, from September 2019 to May 2022, who provided informed consent to participate.

**Results** Even dividing the analysis of increasing the pure tone average (PTA) or speech recognition threshold (SRT) between the difference into initiation of the salvage therapy in up to 15 days of the sudden deafness event, and between 15 days and 3 months of the event, we did not find any difference in hearing improvement.

**Conclusion** Intratympanic corticosteroid therapy is prescribed when conventional therapy fails or when there is a limitation to the use of corticosteroids due to the presence of systemic disorders. As such, new drugs, such as bethametasone, are studied and show promising results.

## Introduction

In the United States, there are approximately 66 thousand new cases of Sudden sensorineural hearing loss (SSNHL) per year. 1 2

This condition is defined as a rapid sensation of hearing loss in one or both ears. The audiometric criterion is a decrease in hearing of at least 30 dB, affecting 3 or more consecutive frequencies compared with the opposite ear's thresholds, and occurring within 72 hours to several days. Also, it is often but not always accompanied by tinnitus and vertigo. According to the National Institute on Deafness and Other Communication Disorders (NIDCD), 3 SSNHL can have multifactorial etiologies, such as inflammation caused by viral infections, immune responses, and vascular insufficiency, as well as many unidentified causes. These factors may explain the high degree of variability in the prognoses. When the cause is not identified, despite adequate investigation, an idiopathic etiology will be considered. Although several etiologies have been postulated, most treated cases are considered idiopathic SSNHL, with only 10% of the cases being identified. 4

Despite the importance of studying the diagnosis protocol, this is not the goal of the present study. We are of the opinion that clinicians should refer to otolaryngologists to proceed with the Weber and Rinne test using a tuning fork, 5 6 and that audiometry should also be performed within 14 days of symptom onset to confirm the diagnosis of SSNHL.

Whenever possible, clinicians should prioritize the request of a magnetic resonance imaging (MRI) scan or, at least, an auditory brainstem response, to identify the retrocochlear pathology and exclude acoustic neurinoma as an etiology, which has a rare incidence of 1:100 thousand habitants per year. 7

Based on the psychological impact that sudden hearing loss can have on patients, the present study intends to advance research on new treatments to improve hearing recovery and patient quality of life.

Much of the literature indicates that the rates of spontaneous recovery (both partial and total) for SSNHL range from 32 to 65%. Most cases resolve within 2 weeks after onset. 8 9 10 11 The only effective treatment using medications reported is systemic high-dose corticosteroid therapy; 4 12 however, despite lasting 2 weeks, the prognosis is not satisfying for some of the patients. 13 For these patients, as well as for those with a contraindication to oral corticosteroids, salvage intratympanic (IT) corticosteroid therapy is mostly indicated, 14 15 even though a metanalysis 16 has reported no differences in the therapeutic outcomes of oral, IT, or the combined treatment.

Furthermore, the IT treatment presents several advantages ( Table 1 ), 17 such as:

**Table 1:** Advantages and disadvantages of intratympanic steroid therapy

Advantages	Disadvantages
Low degree of difficult	Invasive procedure
Outpatient procedure	Risk of tympanic membrane perforation
It can be administered shortly after diagnosis	Pain
Relatively painless	Vertigo (usually temporary)
Can be used in patients to whom oral corticosteroids are contraindicated	Hearing loss risk in case of trauma in the ossicular chain
Higher concentration of corticoids in the cochlear fluid, as detected in research	Risk of otitis media

- The procedure is well tolerated and easy to administer under local (topical) anesthesia.
- The use of general anesthesia is avoided.
- The concept of IT infiltration is easy to understand: a high concentration of a drug is injected into the perilymphatic cochlear fluid.

The purpose of the present work is not to describe the protocol of investigative exams used by our group, but to evaluate the effects of betamethasone in the treatment of SSNHL, used as salvage therapy for IT infiltration, and to correlate the results with the factors for better prognosis.

The first study that found an improvement in hearing in patients receiving oral steroids, published in 1980 by Nadol and Wilson, 18 showed a rate of 78% of improvement, compared to the rate of 38% for placebo.

Other recommended treatment options are antiviral agents, hyperbaric oxygen therapy, plasma volume expanders, carbogenic therapy, vasodilator agents, and diuretics. In some cases, middle-ear surgery for fistula repair is also necessary. At the end of the treatment, patients with partial or no hearing recovery and the persistence of tinnitus or vertigo will require multidisciplinary management, including psychotherapy and audiological rehabilitation.

Regarding medication, the currently accepted gold standard for the treatment of sudden hearing loss is oral steroid therapy, with prednisolone (in doses of 1 mg/kg) is the most used drug. The treatment using IT corticosteroids is well recognized, with positive evidence worldwide, but it remains controversial in the literature concerning the type of corticosteroid, the dosage, and the treatment protocol. 19

In the literature, IT steroid infiltration is recommended as salvage therapy when patients present incomplete recovery after therapy with oral steroids for approximately 3 weeks after the onset of symptoms, or in cases of patients with any comorbidity that contraindicates the use of oral corticosteroids 12 15 20

This therapy aims to reduce inflammation in the inner ear and help to inhibit the injury to cochlear hair cells with a considerably higher concentration of steroids inside the perilymph and inner ear compared with oral systemic administration in guinea pigs; however, IT steroids showed no considerable corticosteroids absorbed into the systemic circulation. 21

In the literature, there is a variability in terms of drug selection, the proposed concentration of steroids, and posology using dexamethasone with dosages of 4, 10, or 24 mg/mL, or methylprednisolone 30 to 40 mg/mL delivered using needle perforation, myringotomy or, in some cases, via tympanostomy tube. 22 But there is only one study 23 about the use of betamethasone for IT salvage therapy as an alternative treatment for SSNHL. Betamethasone is considered a potent corticosteroid, with the same plasma half-life of -300 and 300 minutes, and anti-inflammatory potential of -30 and 30 when compared to dexamethasone. However, the ampoules contain a dose of betamethasone of 7 mg, while in Brazil commercial dexamethasone has a maximum concentration of 4mg/mL. As such, it would be necessary to manipulate the dosage of dexamethasone proposed by international protocols to reach 24 mg, which would lengthen the time until the start of the treatment. Inserting the methyl group at the 16-beta position led to the creation of betamethasone, which is 1.2 times more active and potent than dexamethasone. 24

The similar chemical configuration of these two substances can influence the biopharmacological activity. This also happens with many other chemical compounds with similar structures, in which only the orientation of a group is changed. In this case, the CH3 method, or adding other chemical elements, if it manages to partially or greatly alter its pharmacological behavior. The potency of both substances is very similar. They are still 20 times more potent than hydrocortisone, and 5 to 7 times more potent than prednisone. However, some authors 25 consider betamethasone slightly more potent than dexamethasone.

According to the literature, the recovery prognosis is dependent on several factors that will also be analyzed in the present study, including the degree of hearing loss, patient age, and time between symptom onset and the start of the treatment.

The Siegel criteria 26 are used to quantify the ﬁnal hearing outcomes and absolute hearing gain, and they are as follows:

- Complete recovery -final pure tone average (PTA) threshold better than 25 dB
- Slight recovery - final PTA threshold between 25 and 45 dB.
- Partial recovery - gain of 15 dB.
- Poor recovery - gain of 15 dB with and a final PTA threshold worse than 45dB.

The decision to perform salvage IT steroid therapy is based on the amount of persistent hearing loss following the initial therapy, patient preference, along with the doctor's opinion. The risks and benefits of this treatment should be taken into consideration as well.

All the patients in the present study received an initial or concomitant oral treatment, which included steroids and other medications, with a variation among the cases, considering the protocol of their previous treatment center. The decision to initiate steroid salvage therapy before the end of the oral treatment is dependent on the level of hearing loss. In cases in which the initial PTA was worse than 45 dB, the IT administration of steroids was initiated at the first appointment. When the initial PTA was better than 45 dB, the IT administration of steroids was proposed as a salvage therapy, if there was partial or no response at the end of the oral treatment, considering that the deadline for this salvage therapy is of up to 3 months after the first symptoms. However, in the literature, we found a deadline of up to 1 month after the event.

The procedure was performed every 3 days, for 3 meetings. One week after treatment completion, audiometry was performed and compared with the preinjection values of PTA and speech recognition threshold (SRT) to evaluate if there was an improvement or not. In cases of partial improvement, the 3 applications, 1 every 2 days, were repeated and, after 1 week, a new audiometry was performed.

The salvage therapy ends when the audiogram no longer presents improvement, and, in the present study, this occurred after a maximum of 6 injections.

Hearing improvement was defined as a decrease of 15 dB or more in the PTA at the four frequencies (0.5, 1, 2, and 3 kHz) after the final treatment. Then, the PTA threshold difference was analyzed at each frequency. Therefore, the criteria used to define refractory SSNHL was fewer than 15 dB in PTA gain. 26

Patient characteristics, including sex, age, and time until the IT steroid treatment are summarized in Table 2 .

**Table 2:** Descriptive data stratified in terms of improvement

Variable	Total (N = 37)	Improvement (n = 28)	No improvement (n = 9)
** Laterality**			
Right	21 (56.8%)	15 (53.6%)	6 (66.7%)
Left	16 (43.2%)	13 (46.4%)	3 (33.3%)
**Sex**			
Female	10 (27.0%)	8 (28.6%)	2 (22.2%)
Male	27 (73.0%)	20 (71.4%)	7 (77.8%)
** Age (years)**	51.0 (37.0–61.0)	51.0 (36.8–61.2)	45.0 (43.0–61.0)
** Time until treatment (days)**	21.0 (7.0–35.0)	17.5 (7.0–31.2)	30.0 (30.0–60.0)
** Baseline** **audiometry (dB)**			
250 Hz	50.0 (30.0–60.0)	47.5 (28.8–60.0)	55.0 (40.0–75.0)
500 Hz	55.0 (35.0–70.0)	52.5 (38.8–70.0)	60.0 (30.0–70.0)
1,000 Hz	55.0 (30.0–70.0)	50.0 (30.0–72.5)	70.0 (15.0–70.0)
1,500 Hz	60.0 (25.0–75.0)	57.5 (25.0–76.2)	60.0 (10.0–70.0)
2,000 Hz	60.0 (25.0–75.0)	57.5 (23.8–77.5)	70.0 (45.0–75.0)
4,000 Hz	65.0 (35.0–80.0)	60.0 (28.8–76.2)	70.0 (60.0–80.0)
6,000 Hz	70.0 (35.0–85.0)	70.0 (30.0–86.2)	75.0 (55.0–80.0)
8,000 Hz	70.0 (50.0–90.0)	70.0 (32.5–90.0)	70.0 (60.0–75.0)
**Pure tone average**			
Pretreatment	55.0 (30.0–80.0)	45.0 (30.0–80.0)	60.0 (10.0–70.0)
Posttreatment	25.0 (15.0–45.0)	25.0 (15.0–40.0)	65.0 (10.0–75.0)
** Etiology**			
Idiopathic	16 (43.2%)	8 (28.6%)	8 (88.9%)
Nonidiopathic	21 (56.8%)	20 (71.4%)	1 (11.1%)

**Note:** Data presented as n (%) or median (interquartile range) values.

The PTAs and SRT levels, before and after the salvage treatment, as well as the number of IT injections, are summarized in Table 3 .

**Table 3:** Multivariate logistic regression with improvement as the dependent variable

Variables	Odds ratio	95% confidence interval	*p* -value
Laterality (left)	0.77	0.10–5.71	0.794
Age (years)	1.03	0.98–1.09	0.274
Time until treatment (days)	1.01	0.99–1.03	0.189
Etiology (idiopathic)	60.60	5.02–5,017	0.011

### Inclusion Criteria

The inclusion criteria were: presence of SSNHL, defined as a sensorineural hearing loss of at least 30 dB at 3 contiguous frequencies over 3 days; age ≥ 18 years; patients who did not respond well to the oral treatment; those who presented a mean PTA (at the frequencies of 500, 1,000, 2,000, and 4,000 kHZ) worse than 30 dB of 15 dB worse than the opposite ear at the end of the oral systemic treatment; patients with a diagnosis of diabetes, renal failure, or any contraindication to the oral therapy; time from hearing loss onset to the start of the treatment longer than 14 days, considering oral corticosteroids as the first treatment when the initial PTA was better than 45 dB; patients to whom the IT steroid treatment was proposed during the first consultation (that is, it was not administered as an alternative treatment), if the initial PTA was worse than 45 dB; and patients without a history of ear diseases, except for Menière disease.

### Exclusion Criteria

The exclusion criteria were: patients with contraindications to the IT administration of steroids, such as those with middle- and outer-ear infections; subjects recently submitted to ear surgery; those with middle-ear pathologies, such as eardrum rupture; patients recently submitted to radiation therapy or chemotherapy; subjects with congenital cochlear malformations or cochlear hemorrhage observed on the radiological examination; recent use of ototoxic medications; patients only treated more than 3 months after the event; pregnant subjects; and patients with glaucoma.

As soon as the MRI scan showed any of the exclusion criteria, such as cochlear hemorrhage, perilimphatic fistula or acoustic neuroma, the patients were removed from the statistical analyses.

### Intratympanic Steroid Therapy

Under otomicroscopy or through a 0-degree endoscope attached to an m-scope, using a cellphone application, after confirming an intact tympanic membrane, the patients were placed in the surgical otological position (affected ear up, with the head rotated 45 degrees toward the healthy ear). Topical anesthesia was applied to the external acoustic canal, and lidocaine 10% pump spray, at 10 mg/dose, was applied in the tympanic membrane. The patients were kept in this position for 5 minutes.

After cleaning the local anesthesia using a surgical stylet and cotton through the posteroinferior quadrant of the tympanic membrane, a single myringotomy was performed using 25-G spinal needles, and between 0,5 and 1 mL was slowly injected until it filled the middle ear, which was enough to see overflow in the external auditory canal. During this procedure, the patients were instructed to avoid moving, with the head tilted 45 degrees towards the healthy side, and avoid swallowing for 2 min. During the procedure, most of the patients, reported tasting the medication in their mouth, as it reaches the eustachian tube and the oropharynx. Also, some reported dizziness as the medication filled the tympanic cavity. After the procedure, the patients were instructed to protect the eardrum until the last meeting, when the doctor ensured there was no residual eardrum perforation.

## Results

As shown in Table 2 , from the sample of 37 patients, 28 showed improvement (75,7%) with the salvage therapy, while 9 patients did not (24%); considering the PTA, 56% had the right ear affected and 43.2%, the left. Also, 73% of patients were male, with a median age of 51 years. The median time until the initiation of the salvage therapy with the application of IT corticoid was of 21 days, and 56.8% had the etiology of the deafness suddenly identified, while in 43.2% it was defined as idiopathic. The median PTA preinfiltration was of 55 dB, and the postinfiltration value was of 25 dB.

Table 3 shows the analysis of each dependent variable to obtain hearing improvement after the IT therapy. Factors such as sex, age, and laterality were not statistically responsible for the hearing improvement outcome. Among the patients who received IT injection therapy, the time of treatment initiation was not a dependent variable for success, with no demonstration of statistical significance up to the 3 months stipulated as the time limit for corticosteroid application.

In Table 3 , we also divided the time to start the treatment with IT corticosteroid between up to 15 days and over 15 days from the sudden deafness event, with no statistically significant difference between the results, despite demonstrating a tendency toward better outcomes when treatment started earlier, with a higher percentage of improvement (32.1%) over the patients with no improvement (11.1%). On the other hand, in those with > 15 days until the start of the treatment, the chance of not presenting improvement was higher (88.9%), while 67.9% presented improvement.

Considering all patients, Graphic 1 shows that the greatest PTA difference in improvement occurred in the low frequencies, with 8 kHz being practically unaffected by the therapy, and the vast majority having an improvement in the 7-kHz frequency.

**Graphic 1:**
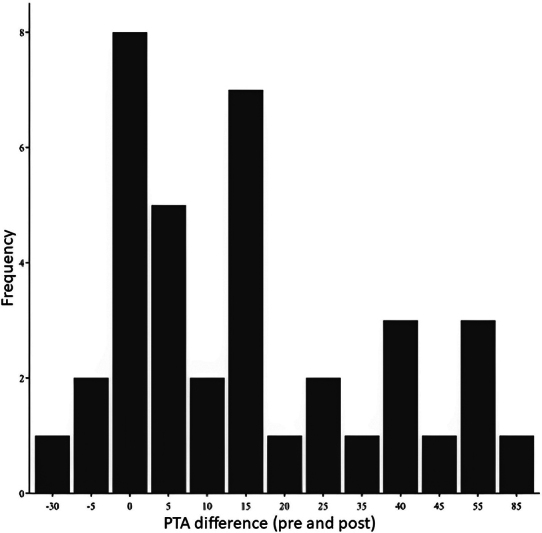
The difference in PTA pre- and postsalvage therapy of the 37 patients.

According to Graphic 2 , hearing improvement occurs when there is a difference greater than 15 dB between pre- and post-IT infiltration levels, which is concentrated in frequencies lower than 4 kHz.

**Graphic 2:**
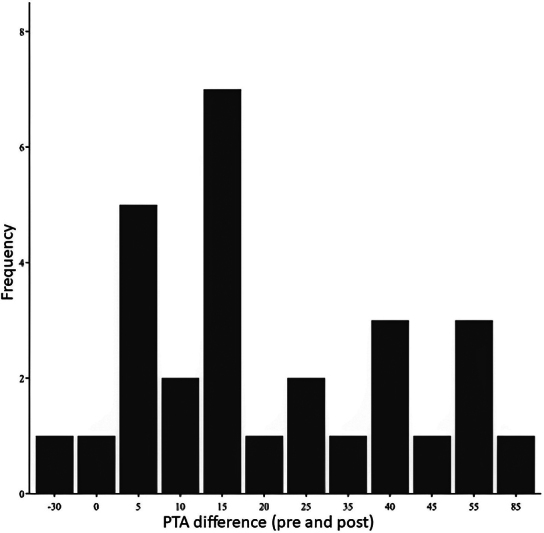
The difference in PTA pre- and postsalvage therapy of the patients who presented improvement.

Graphic 3 shows the PTA median graphs before and after IT therapy, demonstrating a decrease in their values.

**Graphic 3:**
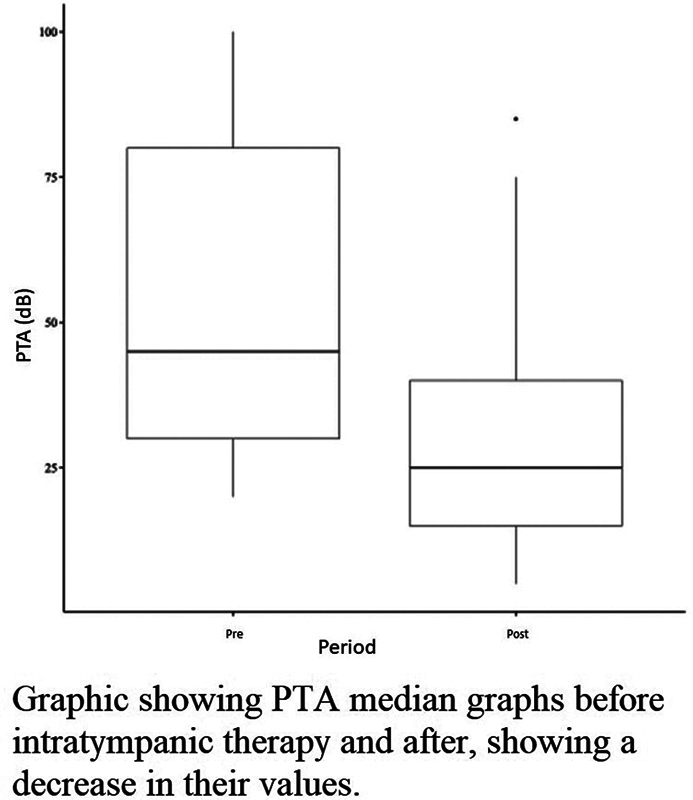
Pre- and posttherapy results of the patients who presented improvement.

## Complications

No severe adverse effects were noted in any of the patients in the present study. Among the final number of 37 patients who received ITS treatment, minor side effects such as local pain at the moment of medication injection was felt by 9 patients, and transient dizziness which lasted for 1 minute on average was felt by 18 of them. One of the patients had dizziness lasting more than 1 minute, described as dizziness, followed by imbalance. This last one felt the symptoms for about 3 hours, which then vanished after taking 1 tablet of symptomatic medication, such as dimenhydrinate. No permanent tympanic membrane perforation was observed at the end of the follow-up, at the last audiogram.

## Statistical Analysis

After data entry, we performed the statistical analysis; the continuous variables were expressed as median and quartile values, And the categorical variables, as relative and absolute frequencies. Table 2 shows the stratification of the sample according to the degree of improvement.

To assess the factors associated with hearing improvement, logistic regression models were used, with the dependent variable in binary format (improvement or not), and categorized as mild, partial, or complete improvement. The sex, age, laterality, and time from onset of symptoms to treatment initiation were analyzed as potential predictors.

All tests were two-tailed and final *p* -values below 0.05 were considered statistically significant. All analyzes were conducted using the R (R Foundation for Statistical Computing, Vienna, Austria) software.

## Discussion

Although SSNHL will often spontaneously improve without any treatment, directed therapy against its known causes and corticosteroid therapy, either systemic or IT, for the idiopathic condition are the mainstays of these patients' care. The possibility of hearing improvement makes this a reasonable treatment, considering its profound impact on quality of life.

The mechanisms through which the drugs act have not been entirely understood, but it is thought that they reduce cytotoxic immune response through their potent antinflammatory effect. They may also increase microvascular blood flow. 27 28

The prognosis for hearing recovery using IT therapy was based on several factors, including duration and degree of deafness, age, starting time of oral medication use, and starting time of IT salvage therapy.

In the present study, even dividing the analysis of increasing the PTA or SRT into time until treatment initiation of the salvage therapy up to 15 days of sudden deafness event, and between 15 days and 3 months of the event, there was no difference in hearing improvement.

As shown in Table 4 , we believe the reason for the lack of statistical difference was due to the prescription of IT corticosteroids within 15 days of sudden deafness in patients who had their first consultation with PTA results worse than 40 dB, which were already considered the worst. However, in general, in patients with early onset of treatment (< 15 days after sudden deafness), the results of the therapy tended to be better. This lead us to believe that a statistical difference with better results from early therapy could be reached if the present study had a larger number of patients to evaluate during follow-up, or if the patients who received IT corticosteroids in the first medical appointment, associated with oral treatment, had the same median SRT as those who received it after finishing the oral treatment.

**Table 4:** Association between time until treatment and outcome

Variables	Total (N = 37)	Improvement (n = 28)	No improvement (n = 9)	*p* -value
Time until treatment: n (%)				0.393
≥ 15 days	27 (73.0%)	19 (67.9%)	8 (88.9%)	
< 15 days	10 (27.0%)	9 (32.1%)	1 (11.1%)	

In studies examining the improvement of sudden SNHL by corticosteroid IT therapy, Koo et al. 29 and Liebau et al. 30 reported recovery rates of about 78 and 55% respectively. In the present study, effective results using betamethasone were observed, with a 75.7% recovery rate and a similar median age of the affected patients.

## Conclusion

Intratympanic corticosteroid therapy is prescribed when conventional therapy fails, or when there is a limitation to the use of corticosteroids due to systemic disorders, such as diabetes mellitus or renal failure.

Based on the results of the present article, further prospective studies are needed, with a control group and treatment standardization, to compare the data obtained and the applicability of the proposed therapy and audiological follow-up with acoustic and electrophysiological exams.

It is not simple to prove the effectiveness of this treatment, but the proposed therapy with betamethasone was also administered to patients with long-term SSNHL, of up to 3 months, who were already stable and had no improvement of the SRT and PTA levels, with the results shown being similar to those of dexamethasone. Finally, the medication chosen by us is more accessible and timesaving for patients, given how the dexamethasone dosage proposed in the literature would require pharmacological manipulation.
